# Octopamine enhances learning

**DOI:** 10.1093/nsr/nwae185

**Published:** 2024-05-30

**Authors:** Yvette E Fisher

**Affiliations:** Department of Molecular and Cell Biology and Helen Wills Neuroscience Institute, University of California, USA

Across the animal kingdom, diverse monoamines actuate and modulate neural circuit function. However, measuring the rapid dynamics of these neuromodulators at cellular resolution has historically presented significant challenges. Lv *et al.* introduce a genetically encoded optical sensor for octopamine. They utilize this method to investigate the function and dynamics of octopamine signaling during aversive learning in *Drosophila*. They find that octopamine signaling enhances aversive odor learning through its interactions with dopamine. This new GRAB_OA_ sensor will facilitate *in vivo* characterization of the dynamics of this monoamine in diverse biological processes [1].

Octopamine is a monoamine found across invertebrate phyla that is analogous to vertebrate norepinephrine. In fact, octopamine was discovered in the salivary gland of octopuses, giving it its charismatic name [2]. Octopamine is an important signaling molecule that has been implicated in diverse processes ranging from social communication to flight behavior [3,4]. In insects, octopamine is implicated in associative learning in a higher order odor center named the mushroom body [5].

**Figure 1.**
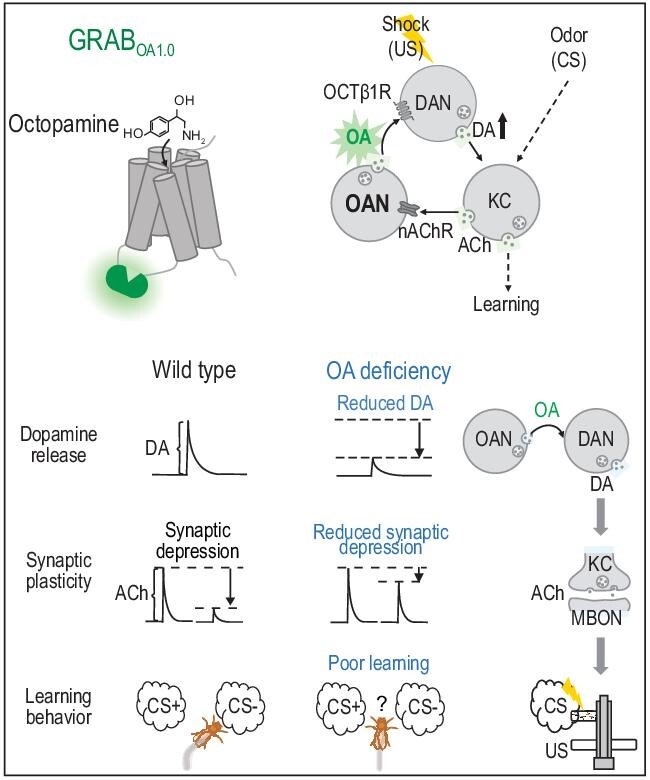
Schematic illustration of GRABOA1.0 design and its application in sensing dynamics of octopamine signaling during aversive learning in *Drosophila*.

First, the authors created a sensor that is sensitive and specific for octopamine—GRAB_OA1.0_ (OA1.0) (Fig. 1). They design a genetically encoded optical sensor using the G protein-coupled receptor activation-based (GRAB) approach [6]. The Octβ2R octopamine receptor sensing region was coupled to circularly permuted enhanced green fluorescent protein so that when octopamine binds fluorescence increases. This approach is compatible with two-photon microscopy, a modality these authors use extensively to measure physiological octopamine release. Importantly, GRAB_OA1.0_ has negligible interactions with endogenous G protein signaling and is specific for octopamine over tyramine, a similar chemical that is also present in the invertebrate nervous system [7].

Next, the authors investigate octopamine dynamics in the *Drosophila* mushroom body. In this concise circuit that mediates associative learning, kenyon cells (KCs) carry odor signals and synapse onto mushroom body output neurons (MBONs). The KC→ MBON synapse is plastic [8]. Synaptic strength is modulated by dopaminergic reinforcement to teach the association between specific odors and approach or avoidance behavior [8]. The mushroom body is additionally innervated by other monoamines that are poised to shape plasticity [9], including octopamine.

To study aversive learning, Lv *et al.* use a conditioning paradigm where a cohort of flies experience an odor paired with an electric shock and another odor without a shock. After a short delay, memory is assayed by testing how much the flies prefer the ‘unpaired’ odor [10]. Flies lacking octopamine synthesis showed impaired learning, consistent with a previous report [11]. Parallel measurements using two-photon imaging of GRAB_OA1.0_ found that octopamine release can occur in response to either odor or shock. Surprisingly, both responses require intact KC neuronal activity. Given that KC cell bodies show no obvious shock responses, this result underscores the complexity of recurrent signaling in this circuit. Next, to test whether learning deficits are accompanied by altered plasticity the authors use GRAB_ACh_ [12] to show that associative synaptic depression of KC synapses is reduced when octopamine neurons are silenced.

How do octopamine neurons influence aversive learning? These authors present evidence that octopamine neuron activity enhances the response of dopamine neurons to electric shock. Enhancement of this dopamine ‘teaching’ signal requires the octopamine receptor Octβ1R. Moreover, flies with Octβ1R knocked down in dopaminergic neurons have learning deficits. Interestingly, a similar circuit logic, but using a different octopamine receptor, has been proposed for appetitive learning [13]. Taken together, this study generated an extremely useful new sensor and clarified how octopamine and dopamine signaling interact to shape aversive learning.

In future work it will be interesting to clarify the specific contributions of distinct octopamine cell types to the learning process and to examine octopamine's involvement in brain regions beyond the mushroom body. Furthermore, this sensor could enable the study of mechanisms that shape octopamine synaptic release and re-uptake, a topic that is unresolved, as a dedicated octopamine transporter has not been identified in *Drosophila* [14]. This sensor can also be applied beyond *Drosophila*, such as in mammals, where octopamine is classified as a trace amine with a potentially important, yet understudied, role in regulating monoaminergic signaling [15]. This sensor creates the opportunity to monitor octopamine dynamics in a wide range of species. In conclusion, the discovery in this work adds to a growing recognition that complex interactions among multiple neuromodulatory systems tunes plasticity, instructing when and how to learn. The generation of this sensor will enable future studies that continue to explore this fascinating topic and the roles of octopamine in diverse biological processes.
